# Dead Tumor Cells Expressing Infectious Salmon Anemia Virus Fusogenic Protein Favor Antigen Cross-Priming *In Vitro*

**DOI:** 10.3389/fimmu.2017.01170

**Published:** 2017-10-09

**Authors:** Jonathan Morales, Carlos Barrera-Avalos, Carlos Castro, Stephanie Castillo, Claudio Barrientos, Claudia Robles-Planells, Ximena López, Ernesto Torres, Margarita Montoya, Marcelo Cortez-San Martín, Denise Riquelme, Alejandro Escobar, Ricardo Fernández, Mónica Imarai, Daniela Sauma, Leonel E. Rojo, Elias Leiva-Salcedo, Claudio Acuña-Castillo

**Affiliations:** ^1^Centro de Biotecnología Acuícola, Departamento de Biología, Facultad de Química y Biología, Universidad de Santiago de Chile, USACH, Santiago, Chile; ^2^Instituto de Investigación en Ciencias Odontológicas, Facultad de Odontología, Universidad de Chile, Santiago, Chile; ^3^Departamento de Salud, Universidad de Los Lagos, Osorno, Chile; ^4^Departamento de Biología, Facultad de Ciencias, Universidad de Chile, Santiago, Chile; ^5^Departamento de Biología, Facultad de Química y Biología, Universidad de Santiago de Chile, USACH, Santiago, Chile

**Keywords:** infectious salmon anemia virus, cross-priming, antitumor immune response, b16 melanoma, cell fusion

## Abstract

Antigen cross-presentation is a crucial step in the assembly of an antitumor immune response leading to activation of naïve CD8 T cells. This process has been extensively used in clinical trials, in which dendritic cells generated *in vitro* are loaded with tumor antigens and then autotransplanted to the patients. Recently, the use of autologous transplant of dendritic cells fused with dying tumor cells has demonstrated good results in clinical studies. In this work, we generated a similar process *in vivo* by treating mice with dead tumor cells [cell bodies (CBs)] expressing the fusogenic protein of the infectious salmon anemia virus (ISAV). ISAV fusion protein retains its fusogenic capability when is expressed on mammalian cells *in vitro* and the CBs expressing it facilitates DCs maturation, antigen transfer by antigen-presenting cells, and increase cross-presentation by DCs *in vitro*. Additionally, we observed in the melanoma model that CBs with or without ISAV fusion protein reduce tumor growth in prophylactic treatment; however, only ISAV expressing CBs showed an increase CD4 and CD8 cells in spleen. Overall, our results suggest that CBs could be used as a complement with other type of strategies to amplify antitumor immune response.

## Introduction

Dendritic cells are the most important antigen-presenting cells (APCs); they are present at trace levels in the tissues and stand for less than 0.5% of circulating leukocytes. These cells activate naïve CD8 T cells by delivering antigens through cross-priming and this particular characteristic has been used in cancer immunotherapy clinical trials (1). Immunotherapy using dendritic cells *in vitro* requires a large amount of cells, these are obtained by differentiating monocytes or CD34+ progenitors with granulocyte-macrophage colony-stimulating factor and IL-4 (2). These cells can be loaded with tumor antigens and multiple techniques have been used for this purpose, including tumor-extracted RNA transfection, pulsing with tumor lysates, apoptotic body induction, peptides, tumor-derived exosomes, and heterokaryon-induction from tumor-dendritic cell fusion (3).

The antigen source for dendritic cells loading is important in the antitumor response; in prophylactic treatments *in vivo*, complete cell structures like exosomes induce a potent antitumor response (4, 5). The cytotoxic effect over the tumors induced by chemotherapeutic agents (6) generates an immunogenic cell death (ICD) and is linked with an antitumor immune response (7). In this context, animals inoculated with dying tumor cells undergoing ICD (induced by oxaliplatins) elicit tumor-specific immune responses protecting the animals against subsequent tumor challenges (8).

Immunogenic cell death enhances exogenous antigen cross-presentation in the context of MHC class I (9); in this regard, Li et al. (10) showed that starved HEK 293T cells, expressing the chicken egg albumin (OVA), undergoes macro-autophagy, presenting an enhanced OVA cross-presentation to CD8+ T cells (11). Furthermore, the inhibition of autophagy reduced the epitope-specific CD8+ T cell antigen cross-presentation (11).

Virotherapy induces tumor cell death and present potential advantages over conventional treatments (12). The type of cell death induced by this therapy is important and the mechanism is not well understood. In this regard, some types of virotherapy can generate ICD (13), through the induction of *in vivo* fusion between tumor cells (14). On the other hand, Hoffmann et al. (15) demonstrated that only the use of viral fusogenic membrane glycoproteins (FMGs) are enough to induce tumor cells fusion leading to a potent and localized tumor size reduction. Furthermore, B16 melanoma expressing the fusogenic membrane protein G from the vesicular stomatitis virus (VSV-G) improve the efficacy of weak allogeneic vaccines (16). These data suggest that ICD induced by FMGs could be a mechanism to improve tumor regression by increasing cross-priming.

In the infectious salmon anemia virus (ISAV), a member of the influenza virus family (17), the infection is initiated by receptor binding and internalization in endosomes; the viral and endosomal membrane is fused by a mechanism mediated by the ISAV fusion protein. In this context, ISA fusion protein expressed in tumor cell bodies (CBs) (dead cells) might be a good candidate to mediate the fusion between the CB and the phagosome or cellular membranes of the APCs, thus delivering antigens to the cytoplasm enhancing cross-priming. Here, we report that the prophylactic antitumor treatment using CBs, independent of the expression of ISAV fusion protein suggesting that CBs can be used as a complement with other antitumor strategies.

## Materials and Methods

### Animals and Cell Cultures

Eight- to ten-week-old C57BL/6J (H2b) were obtained from the Universidad de Santiago de Chile animal facility. The animals were fed *ad libitum* with a 12/12 h light/dark cycle. All procedures were conducted in accord to guidelines on the recognition of pain, distress, and discomfort in experimental animals described by Morton and Griffiths, except for temperature evaluation (18). Protocols were reviewed and approved by the Ethics Committee of the Universidad de Santiago de Chile.

HEK293 (kindly provided by Dr. Andres Stutzin), MDCK (kindly provided by Dr. Monica Imarai), Raw264.7 (kindly provided by Dr. Maria Rosa Bono), and murine melanoma B16 (kindly provided by Dr. Flavio Salazar) cell lines were cultured in Dulbecco’s modified Eagle’s medium. Media was supplemented with 10% fetal bovine serum, 100 U/mL penicillin, and 100 µg/mL streptomycin and cells were kept at 37°C in a humidified atmosphere under 5% CO_2_. Mouse bone marrow-derived dendritic cells (BM-dendritic cells) were generated as previously described (19).

### ISAV Fusion Protein and Transfections

Fusion protein sequence was isolated from an ISAV outbreak in Chile, the fusion protein is encoded in the segment 5 of the ISAV genome. The ISAV fusion protein gene sequence was synthesized by Genscript (NJ, USA) and subcloned from pUC57 using primers containing the sequence for EcoRI and XhoI for pIRES, and HindIII and XhoI for pCDNA3.1.

HEK293, MDCK, and B16 cell lines were transfected with pIRES-ISAV or pcDNA3.1-ISAV using Lipofectamine 2000 (Thermofisher, USA) according to the manufacturer’s recommendations. Stably transfected cells were selected and maintained with 0.4 mg/mL G418.

### CBs Generation

Infectious salmon anemia virus-transfected or wild-type B16 or HEK293 cells were grown until 70% confluence, and then they were washed with PBS and deprived of nutrients by switching culture media to PBS containing 2.5 µg/mL fungizone and 10 µg/mL gentamycin for 1 week at 37°C in a humidified atmosphere under 5% CO_2_. At day 7, the supernatant was centrifuged at 300 *g* and the pellet was stored in PBS at 4°C.

### Cell Fusion Assays

Infectious salmon anemia virus stably transfected HEK293, MDCK, and B16 cell lines were growth at 70–90% confluence. Cell fusion was morphologically evaluated on a light microscope; 10 random field at 20× magnification were captured and analyzed using a CMOS camera (AmScope). To measure cell fusion in MDCK cells, we used CellTracker™ Green (CMFDA), which labels cytoplasm and nuclei, and CellTracker™ Red (CMTPX), which labels preferentially the cytoplasm. Briefly, MDCK cells were grown until 80% confluence, then the cells were incubated for 45 min at 37°C in non-supplemented DMEM containing 20 µM CMFDA or 15 µM CMTPX, washed, and then incubated for two additional hours. These cells were trypsinized and mixed in a 1:1 ratio (5,000 cells in total), and the suspension of mixed cells was seeded on 12 mm round coverslips and cultured for 24 h. The cells were fixed in 1% w/w paraformaldehyde for 10 min at RT and mounted with 10% DABCO (Sigma). The fluorescence images were acquired on a Zeiss LSM510 confocal microscope and processed using the Zeiss LSM 4 Image Browser software.

### Dendritic Cell Maturation

Dendritic cell maturation was induced at day 7 post differentiation by adding 1.0 µg/mL LPS from *E. coli* 026:B6 (Sigma) or CBs in 0.5:1, 1:1, and 2:1 ratios (CBs/dendritic cells) for 24 h. After the challenge, the cells were collected and labeled with the following antibodies: anti-mouse-CD11c-PE (clone N418), anti-mouse-CD86-APC (clone GL1), anti-mouse-MHC class II- FITC (clone M5/114.15.2), anti-mouse/rat CD40-FITC (clone HM40-3), and anti-mouse-MHC class I-FITC (clone AF6-88.5.5.3) (eBioscience). The samples were analyzed by flow cytometry using the Accuri C6 Flow Cytometer (BD Bioscience), and the data were processed with the CFlow Plus software.

### Cross-Presentation in Dendritic Cells

Dendritic cells were stimulated for 24 h with HEK293-OVA or HEK-OVA-ISAV CBs in a 1:1 and 2:1 ratio (CBs/dendritic cells). As a positive control, the cells were pulsed with 5 µM OVA257-264 derivative peptide (SIINFEKL, S7951, Sigma) incubated for 3 h at 37°C in a humidified atmosphere under 5% CO_2_. After this time, cells were labeled with anti-mouse-CD11c and anti-mouse OVA_257–264_ (SIINFEKL/H-2Kb, clone eBio25-D1.16, eBioscience). The cells were analyzed by flow cytometry.

### Cytoplasmic Antigen Transfer Assay

HEK293-ISAV stably transfected or wild-type cells were grown until 80% confluence on 100 mm dishes, incubated with 20 µM CMFDA, and CBs were prepared as described above. Dendritic cells or Raw264.7 cells were incubated with 10 µM CMTPX or 15 µM CellTracker™ (CMRA), respectively, for 45 min, and then they were challenged with fluorescent CBs for 24 h. Cells were fixed in 1% PFA for 10 min and incubated with DAPI (0.5 µg/µL) for 1 min for confocal microscope analysis. For Raw264.7 and DCs, we quantified the CBs by measuring the mean fluorescence intensity in the cytoplasm using ImageJ (20).

### Antitumor Treatment on Melanoma Cancer Model

To assess the effect of immunization with fusogenic CBs, we used a melanoma model (B16 cells). For preventive treatment, C57BL/6J mice were immunized with 100 µL of CBs (from 1 × 10^5^ cells) every 7 days for 3 weeks generated from wild-type B16 or fusion protein ISAV-transfected cells (B16-ISAV). CBs were injected subcutaneously in the abdominal region, far from the tumor growth area. One week after the final immunization, the mice were challenged with 2 × 10^5^ viable B16 cells. In parallel, we evaluated the curative effect of fusogenic CBs on melanoma. Briefly, the mice were injected subcutaneously in the lumbar region with 2 × 10^5^ viable B16 cells (C57BL/6J), and 7, 14, and 21 days after the challenge they were inoculated with 100 µL of CBs (from 1 × 10^5^ cells) generated from wild-type or ISAV-B16 transfected cells. For both treatment protocols, tumor appearance and size were monitored daily, when the tumor reached a volume of 250 mm^3^ (calculated by a semi-sphere formula *V*[mm^3^] = 2/3π*r^3^) mice were sacrificed and the spleen and tumor were removed.

To evaluate different lymphocyte populations, the spleen of treated mice was disaggregated on a 100 µm metal mesh and erythrocytes were eliminated by differential lysis, using ACK buffer (10 mM KHCO_3_, 155 mM NH_4_Cl, 1 mM EDTA, pH: 7.3). For the tumor-infiltrating lymphocyte (TIL), the tumors were cut into small pieces, disaggregated, and suspended using a Tumor Dissociation Kit (MiltenyiBiotec Inc.). Splenocytes and tumor cells were suspended at a density of 1 × 10^6^ cell/mL in cold blocking buffer (IF; 2% FBS in PBS) for 30 min at 4°C. The following antibodies were used to identify the lymphocyte populations by flow cytometry: anti-mouse CD4 FITC (cat. 11-0042) and anti-CD8 Ly-2 PE (cat. 553033) (BD Biosciences).

### Statistical Analyses

Tumor appearance was evaluated by the Kaplan–Meier method. Tumor growth was analyzed by multiple *t*-test followed by Holm–Sidak’s method. Cell fusion induced by ISAV fusion protein and antigen transfer was analyzed using the Mann–Whitney test. The percentage of CD8+, CD4+, Tregs, Th1, and Th17 lymphocytes was analyzed using Kruskal–Wallis ANOVA. For all experiments, statistical differences between groups and treatments were considered significant when *p* < 0.05. All statistical analyses were performed using GraphPad Prim version 5.

## Results

### ISAV Fusion Protein Keeps Its Fusogenic Capability in Mammalian Cells

**Figure 1:**
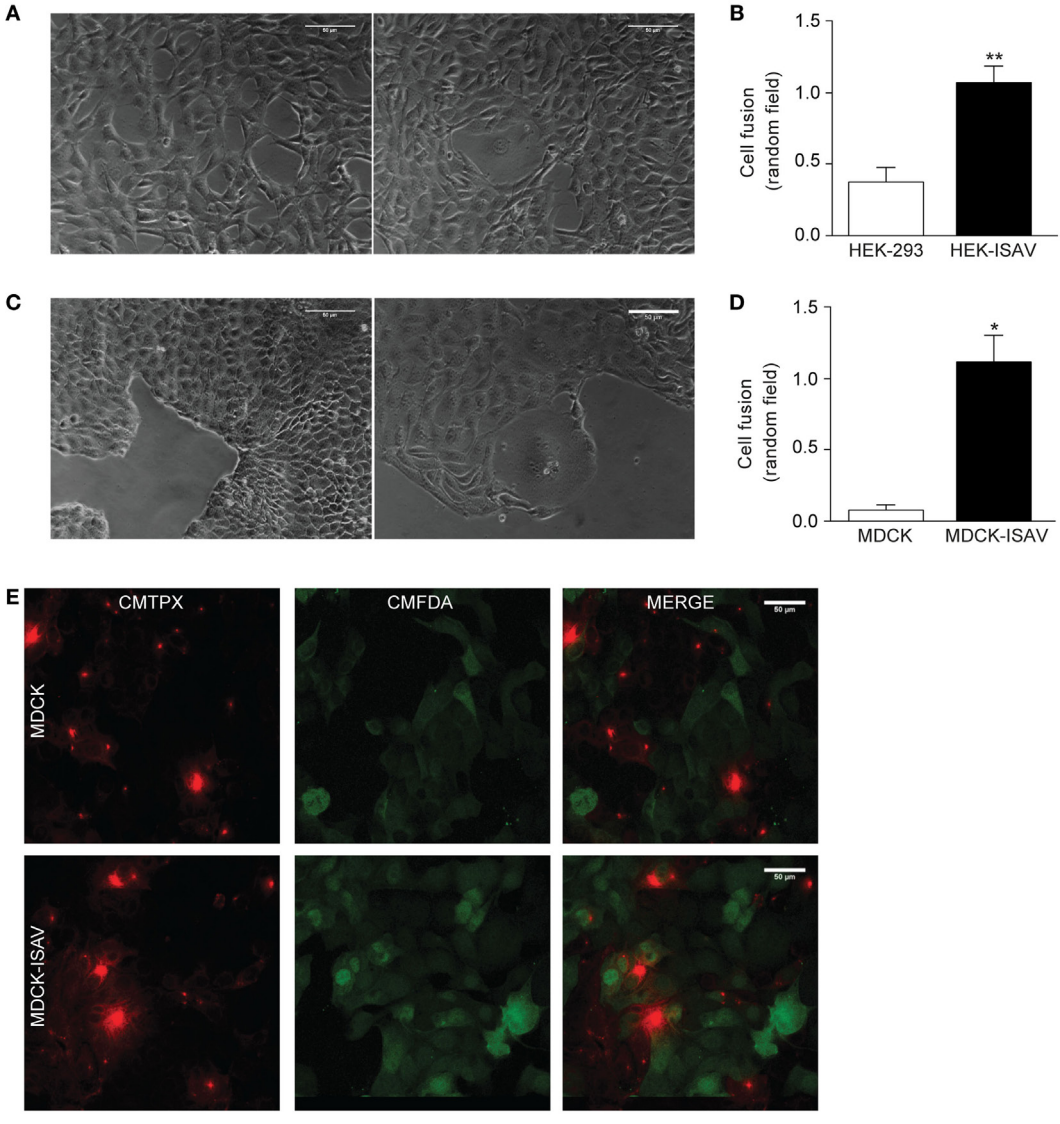
Fusion protein infectious salmon anemia virus (ISAV) induces fusion in HEK293 and MDCK cells. **(A)** Light microscope images of cell fusion in non-transfected (left) or ISAV-transfected HEK293 cells (right), **(B)** quantification of cell fusion of 10 random fields in non-transfected or ISAV-transfected HEK293 cells (*n* = 5). **(C)** Light microscope images of cell fusion in non-transfected (left) or ISAV-transfected MDCK cells (right). **(D)** Quantification of cell fusion of 10 random fields in non-transfected or ISAV-transfected MDCK cells (*n* = 5). **(E)** MDCK cells were labeled with CMFDA or CMTPX separately, and then they were mixed and grown for 24 h. The upper panel shows non-transfected MDCK and the lower panel shows ISAV-transfected MDCK. Statistical analyses were performed using the Mann–Whitney test (*p < 0.05, **p < 0.01).

To determine whether ISAV fusion protein retains its fusogenic activity when expressed in mammalian cells, HEK293 and MDCK cells were stably transfected with the open reading frame of ISAV segment 5, which corresponds to the fusogenic protein (fusion protein ISAV). We found that after 48–72 h post-passage, non-transfected HEK293 and transfected HEK293-ISAV showed morphological changes associated with cell fusion (Figure 1A); however, HEK293-ISAV presents a higher number of fused cells (HEK293 = 0.38 ± 0.23 vs. HEK293-ISAV = 1.08 ± 0.26; Figure 1B). In addition, we evaluated cell fusion in MDCK cells because this cell line has a polygonal morphology with an ordered epithelial monolayer, and the limits between cells are easily distinguishable. We found that ISAV fusion protein induces fusion in MDCK, showing zones with an increased cell size, loss cell limits, and loss of the polygonal shape (Figure 1C). The quantification showed that MDCK expressing ISAV fusion protein showed a higher number of fused cells (MDCK = 0.08 ± 0.083 vs. MDCK-ISAV = 1.13 ± 0.38; Figure 1D).

To test the action of the ISAV fusion protein on cell fusion, MDCK cells were labeled with CMTPX or CMFDA. Non-transfected MDCK cells showed only a single fluorophore labeling, with no overlapping (Figure 1E, top panel). However, MDCK-ISAV showed labeling of both fluorophores, a cytoplasmic red labeling and a nuclear and cytoplasmic green labeling (Figure 1E, bottom panel). These results indicate that ISAV fusion protein allows the transfer of the fluorophores between cells, indicating a process of cellular fusion.

### ISAV Fusion Protein Expression on CBs Allow Antigen Transfer by Increasing Its Internalization in APCs

**Figure 2:**
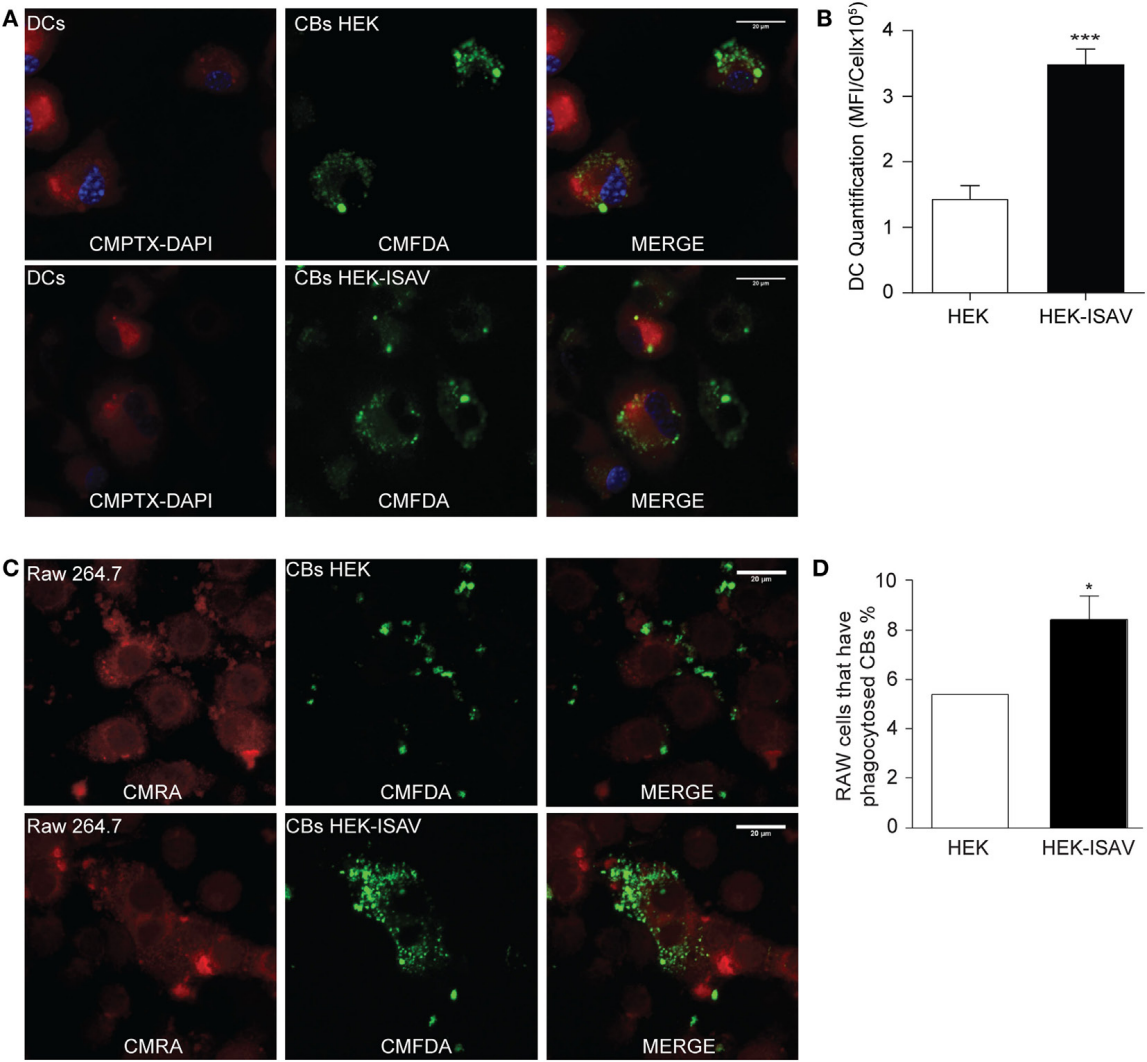
Fusion protein infectious salmon anemia virus (ISAV) facilitates antigen transfer to antigen presenting cells. **(A)** Phagocytosis of fluorescent cell bodies (CBs) by dendritic cells; the top panel shows dendritic cells in the presence of non-transfected CBs; the bottom panel shows dendritic cells in the presence of CBs transfected with ISAV. **(B)** Quantification of mean fluorescence intensity of CBs in the cytoplasm of DCs **(C)** Phagocytosis of fluorescent CBs by Raw.267; the top panel shows Raw.267 in the presence of non-transfected CBs, the bottom panel shows Raw.267 in the presence of CBs transfected with ISAV. **(D)** Quantification of RAW.264.7 cells percent that have phagocytosed fluorescent CBs (*n* = 7). Statistical analyses were performed using the Mann–Whitney test (*p < 0.05, ***p < 0.001).

Once we confirmed that the ISAV fusion protein keeps its fusogenic capability in mammalian cells, we evaluated the difference in the internalization levels of CBs generated from HEK293 and HEK293-ISAV using Raw264.7 and BM-dendritic cells. Both cell types challenged with HEK293 CBs showed low levels of internalization; in contrast, cells challenged with CBs from HEK293-ISAV showed larger numbers of cells associated with CBs (Figures 2A,C). These results show that the ISAV fusion protein expression on CBs derived from HEK293 cells produce an efficient antigen internalization and transfer in both Raw264.7 (Figure 2B) and dendritic cells (Figure 2D) as compared with ISAV non-expressing cells.

### ISAV Fusion Protein Does Not Interfere with Dendritic Cell Maturation and Cross-Presentation Induced by CBs

**Figure 3:**
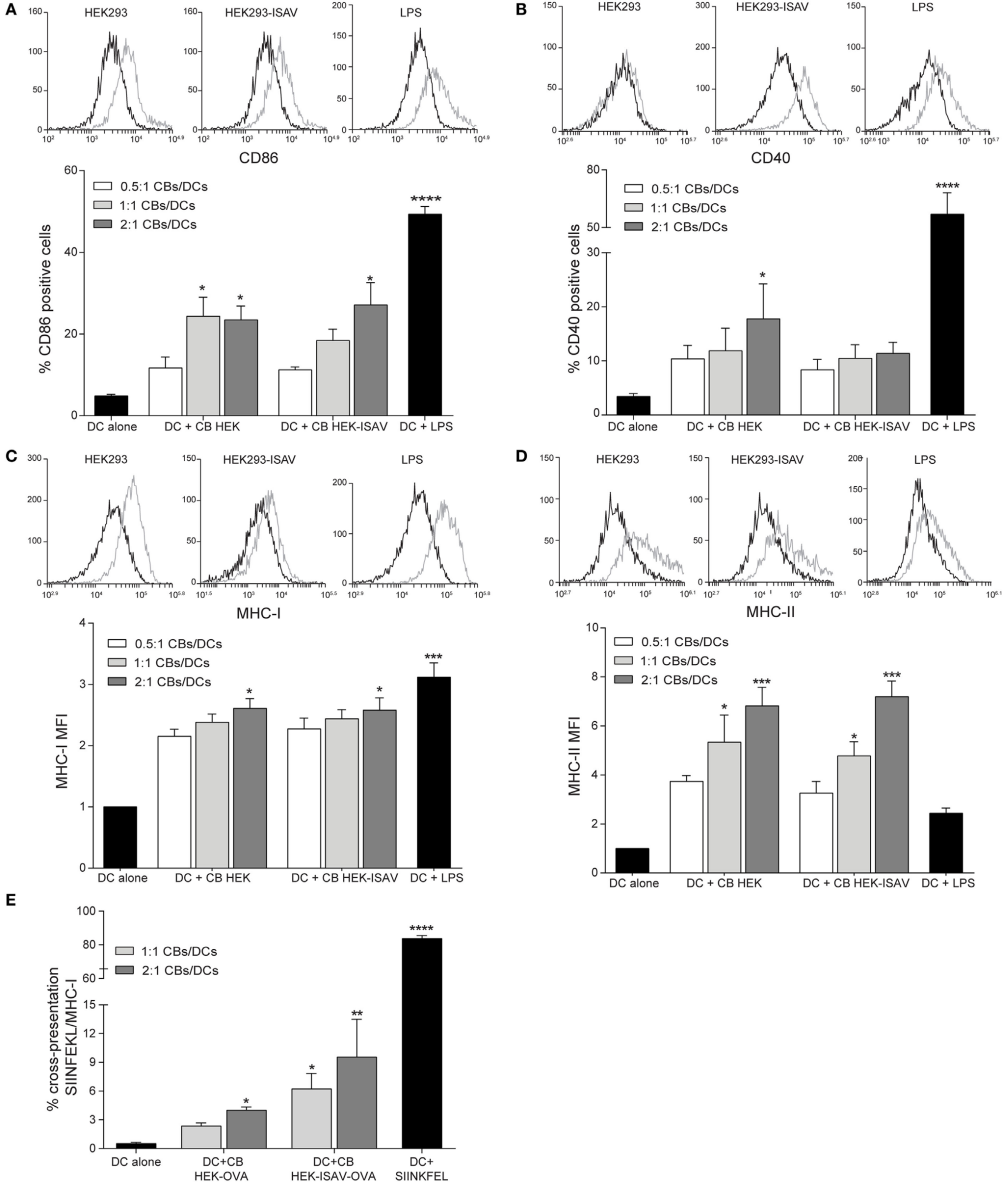
HEK293 cell bodies (CBs) induce dendritic cell maturation. Dendritic cells were challenged with different ratios of CBs derived from non-transfected and infectious salmon anemia virus (ISAV)-transfected HEK-293 cells for 24 h. Maturation markers were measured by flow cytometry, for each marker, a representative histogram of the ratio 1:1 [CBs/DCs] is shown (control, black line; stimulated, gray line). **(A)** % CD86 and **(B)** % CD40 positive cells. **(C)** MHC-I and **(D)** MHC-II mean fluorescence intensity (MFI) (*n* = 5). **(E)** Cross-presentation was measured by the detection of SIINFEKL-MHC-I (*n* = 6). Statistical analyses were performed using Kruskal–Wallis ANOVA (*p < 0.05, ** *p* < 0.005, ***p < 0.001).

To characterize the effect of CBs on dendritic cell maturation, dendritic cells were challenged with different ratios of CBs (0.5:1, 1:1, and 2:1 CBs/DCs). The evaluation of surface markers of maturation showed a low expression of CD86 and CD40 co-stimulatory molecules in non-stimulated dendritic cells (Figures 3A,B, respectively). On the other hand, dendritic cells stimulated with CBs from HEK293 and/or from HEK293-ISAV showed an increase in the expression of CD86 (Figure 3A) and CD40 (Figure 3B), but maturation levels were lower than in dendritic cells stimulated with LPS (positive control). Furthermore, we observed an increase in MHC-I (Figure 3C) and MHC-II (Figure 3D) expression in dendritic cells treated with CBs from HEK293 and HEK293-ISAV, and in the case of MHC-II reaching higher levels than LPS. We did not find differences in dendritic cells maturation with CBs from HEK293 and HEK293-ISAV.

To determine the effect of fusogenic CBs on cross-presentation, we generated CBs from HEK293 that expressed OVA antigen and the fusion protein ISAV (confirmed by RT-PCR). Dendritic cells challenged with HEK293-ISAV-OVA CBs showed a higher level of MHC-I/SIINFEKL than dendritic cells incubated with CBs from HEK293-OVA. However, none of them reached the levels of expression of dendritic cells loaded with OVA257-26 peptide (SIINKFEL) (Figure 3E). Altogether, these results show that the expression of fusion protein ISAV in cellular bodies does not interfere with dendritic cells maturation neither cross-presentation.

### Treatment with CBs Delays B16 Melanoma Tumor Growth

**Figure 4:**
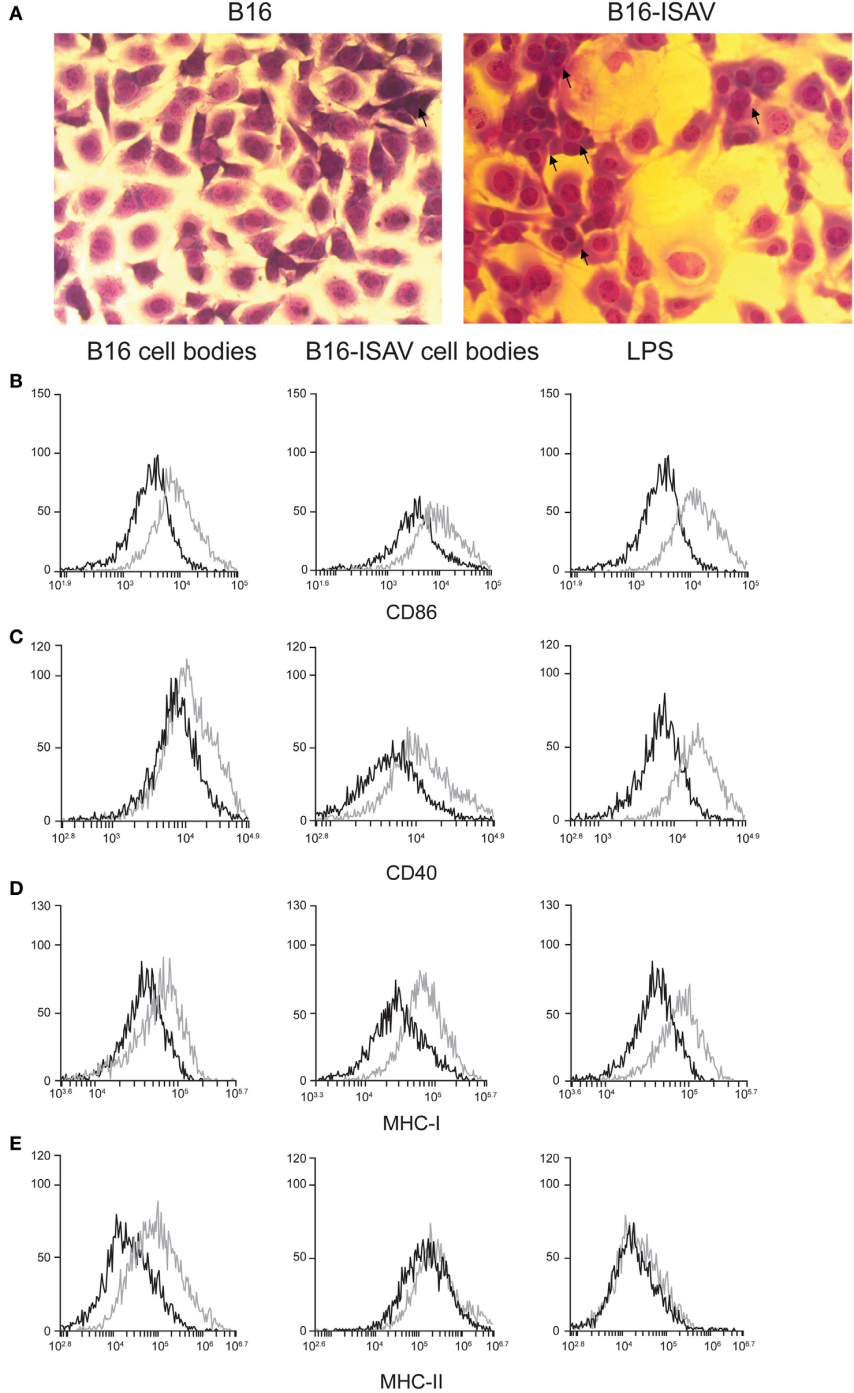
Fusion protein infectious salmon anemia virus (ISAV) induces fusion in B16, and cell bodies (CBs) derived from B16 and B16-ISAV induce dendritic cell maturation. **(A)** Cell fusion was evaluated by transmitted light microscopy and hemacolor stain in non-transfected and ISAV-transfected cells B16 (arrows indicate fused cells). The expression of maturation markers determined by the percentage of **(B)** CD86 and **(C)** CD40 positive cells, and by the mean fluorescence intensity for **(D)** MHC-I and **(E)** MHC-II, induced by CBs was measured in dendritic cells by flow cytometry. Representative histograms of the ratio 1:1 (CBs/DCs) of dendritic cells stimulated with B16 and B16-ISAV bodies (control, black line; stimulated, gray line).

While cellular bodies induce maturation of dendritic cells, we sought to determine whether CBs induce an antitumor immune response when they are used as a treatment. First, we evaluated the cell fusion capability of ISAV fusion protein using a syngeneic melanoma model (B16). In this experiment, we found that ISAV protein induces cell fusion in B16 cells (Figure 4A). Next, we evaluated the dendritic cell maturation in response to different ratios of cellular bodies generated in the B16 and B16-ISAV cell lines, this treatment increases the expression of CD86 (Figure 4B), CD40 (Figure 4C), MHC-I (Figure 4D), and MHC-II (Figure 4E) in dendritic cells in a 1:1 and 2:1 ratio (CBs/DCs) showing that both CBs induced dendritic cells maturation.

**Figure 5:**
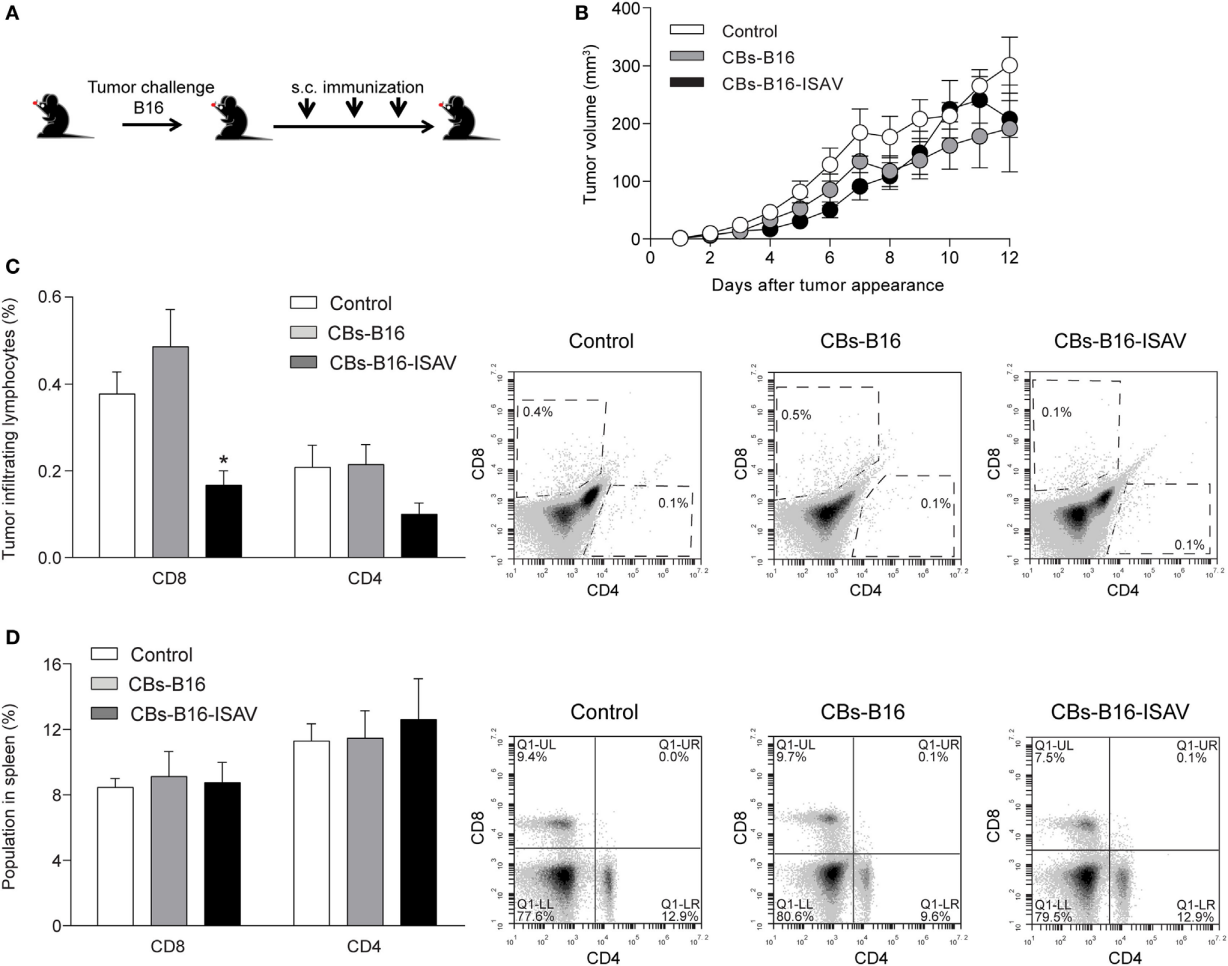
Effect of curative treatment with B16 and B16-ISAV cell bodies (CBs) in C57BL/6, challenged with B16 tumor cells. Schematic representation of the curative treatment. **(A)** C57BL/6 mice were challenged with 2 × 10^5^ viable B16 cells and 7, 14, and 21 days after the challenge they were inoculated with 100 µL of cellular bodies (from 1 × 10^5^ cells) generated from non-transfected or infectious salmon anemia virus (ISAV)-transfected cells. **(B)** Tumor growth after tumor appearance in C57BL/6 mice. **(C)** CD8 and CD4 tumor infiltrating lymphocytes in C57BL/6 mice treated with B16 CBs. **(D)** CD8 and CD4 spleen lymphocytes in C57BL/6 mice treated with B16 CBs. Statistical analyses were performed using Kruskal–Wallis ANOVA (*n* = 6; *p < 0.05).

To determine the effect of CBs on the growth of established tumors, we generated tumors from B16 cells on C57BL/6 mice (Figure 5A). In the B16 melanoma tumor model, the treatment with CBs and ISAV CBs do not change the tumor growth (Figure 5B), with a significant decrease in CD8 TILs (Figure 5C) on B16-ISAV treated mice. To address whether both CBs treatment were linked to a systemic response, we assessed the CD4 and CD8 cell populations in the spleen. We found no changes in CD4 and CD8 populations (Figure 5D), in response to treatment compared to untreated animals.

**Figure 6:**
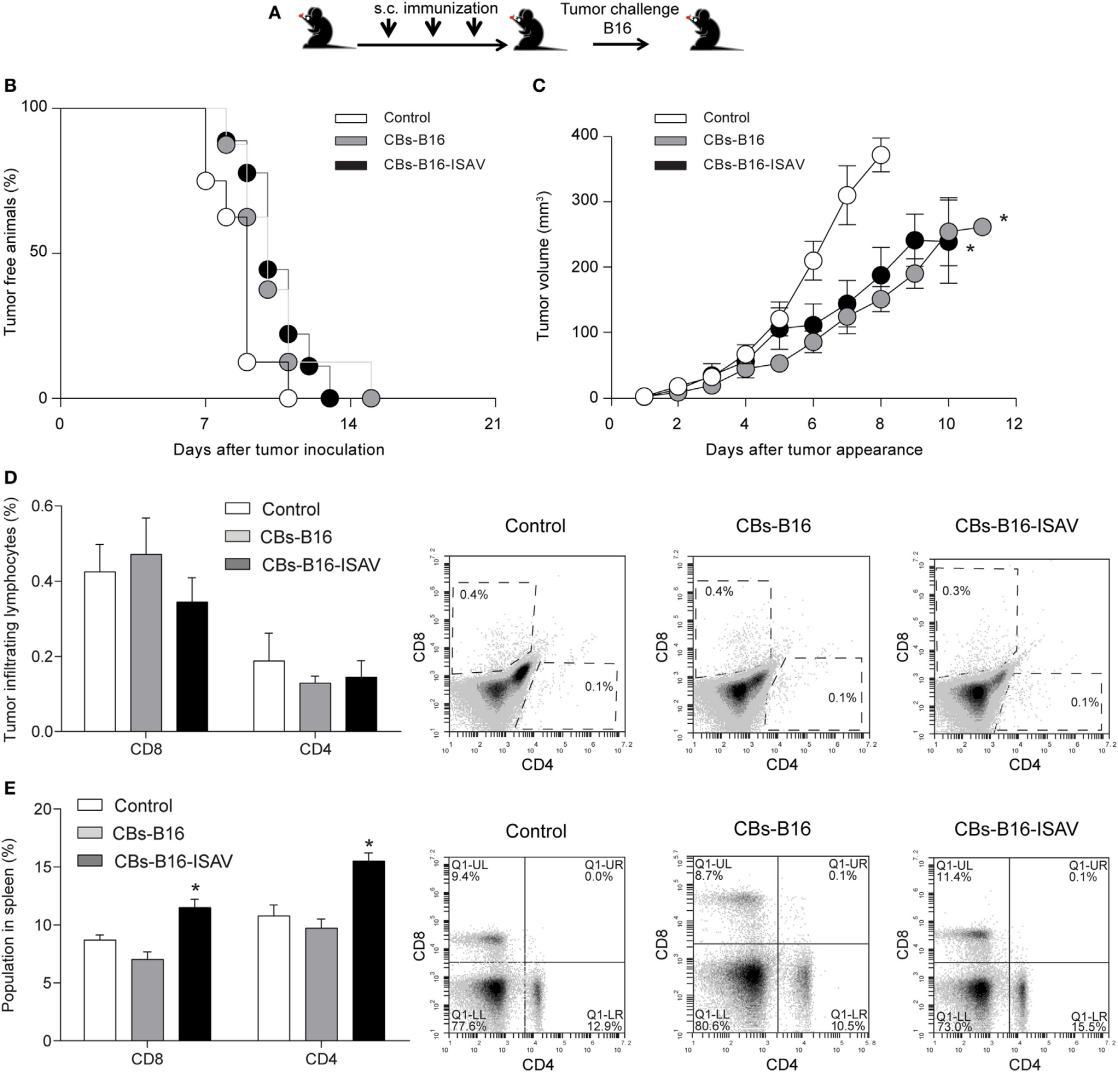
Effect of immunization with B16 and B16-ISAV cell bodies in C57BL/6 mice challenged with B16 tumor cells. **(A)** Schematic representation of the immunization protocol. C57BL/6 mice were immunized every 7 days at 3-week intervals with 100 µL of cellular bodies (from 1 × 10^5^ cells) generated from non-transfected or infectious salmon anemia virus (ISAV)-transfected B16 cells. One week after the final immunization, the animals were challenged with 2 × 10^5^ viable B16. **(B)** Tumor free animals. **(C)** Tumor growth after tumor appearance in C57BL/6 mice. **(D)** CD8 and CD4 tumor infiltrating lymphocytes. **(E)** CD8 and CD4 spleen lymphocytes. Statistical analyses were performed using Kruskal–Wallis ANOVA (*n* = 7; *p < 0.05).

To evaluate whether tumor growth masked the effect of the curative treatment on established tumors, we determine the effect of the previous immunization on tumor development using both types of CBs in C57BL/6 mice (Figure 6A). We found that mice immunized with CBs from B16 and B16-ISAV did not show a delay in tumor appearance (Figure 6B) but instead it showed a delay in tumor growth (Figure 6C). The immunization with B16-ISAV CBs was not correlated with changes in the CD8 and CD4 populations in the tumor (Figure 6D), but it was correlated with an increase of the CD8 and CD4 populations in spleen (Figure 6E).

## Discussion

Immunotherapy is a promising tool for treatments against cancer, numerous studies demonstrated that this type of therapy can prevent or stop tumor growth. Although this therapy has brought satisfactory results, its cost has undermined their clinical use. Recent evidence suggests that chemotherapeutical agents can induce ICD and triggers an immune response, thus participating in the eradication of the tumor. In this work, we tested the effect of antigen-loaded CBs expressing ISAV fusion protein generated *in vitro* (which mimics ICD) as an immunotherapy against melanoma. Using a prophylactic treatment paradigm, we observed a delay in tumor growth. Furthermore, CBs stimulate dendritic cells maturation and induce antigen cross-presentation *in vitro*.

The effective activation of an antitumor immune response requires the delivery of antigens to dendritic cells inducing cross-priming and an efficient activation of reactive T cells. In this context, the use of whole tumor cells as an antigen source offers an advantage by its high diversity of undefined antigens, increasing the chances to mount a robust immune response (21). For instance, dendritic cell vaccines pulsed with apoptotic CBs (derived from whole tumor cells) induce a more efficient antitumor immune response (22) than dendritic cell vaccines pulsed with tumor lysates (23). In this regard, our results show that activated dendritic cells can phagocytize apoptotic CBs, suggesting that apoptotic CBs, as a source of antigens, may improve antigen transference into dendritic cells.

In salmon cells, ISAV-F protein stimulates cell fusion in the absence of accessory glycoproteins (24). Using ISAV-F protein, we observed cell fusion in mammalian cells lines such as HEK293, 4T1 (data not showed), B16 and MDCK. Moreover, cells bodies expressing ISAV-F protein favors antigen delivery into dendritic cells in a mechanism related to the fusion between dendritic cells and CBs membranes [similar to the study by Koido et al. (25)], this is not correlated with differences in dendritic cell maturation and antigen cross-presentation. In contrast, dead cells expressing fusion proteins in its surface display an increased cross-presentation (26). Our result could be explained because the mammals are not the natural hosts of ISA virus and the ISAV-F protein activity could be different at the pH (7.4 vs. 5.4) and temperature (37 vs. 14°C) tested (24).

Our results demonstrated that CBs induce dendritic cell maturation *per se*, this effect could be related to the autophagy induced by nutrient deprivation (27), which induce the expression of High-Mobility Group Box 1 nuclear protein, calreticulin translocation, and the release of degradation products such as ATP (28). All of these components are well-known damage-associated molecular patterns (DAMPs), which activate dendritic cell (29). Our studies indicate that our method to produce CBs induces ICD, and these CBs express DAMPs (Lopez et al., manuscript in preparation).

We observed that prophylactic treatment using CBs from B16 and B16-ISAV generates a delay in tumor growth in B16 melanoma model, but only the treatment with B16-ISAV CBs showed an increase in systemic CD8+ and CD4+ lymphocytes which are associated with an antitumor response. Nonetheless, in both treatments (prophylactic and curative), we did not observe variations in CD8 and CD4 TILs population and we only found a reduction in the CD8 TIL in the curative treatment. The interpretation of these results requires the precise analysis of CD45+ population to determine the extent of CD8+ and CD4+ TILs variations. Additionally, the lack of effect in lymphocytes population may be explained by the timing of the population analysis of the spleen and tumor, these were performed in the late phase of the tumor growth, when the tumor already surpassed the immune system by inducing a tolerogenic response. In this regard, it will be necessary to measure the population at initial stages of growth to obtain the precise kinetic of the populations during the immune response against the tumor.

Altogether, our findings suggest the use of CBs expressing ISAV fusion protein induce dendritic cells maturation *in vitro*, antigen transfer by macrophages and dendritic cells, and an increased cross-presentation by dendritic cells. Despite the delay in tumor growth, this effect is not related to a resolving immune response. Overall, our results suggest that CBs can be used as a complement with other strategies to amplify the antitumor immune response.

## Ethics Statement

All procedures were conducted in accord to guidelines on the recognition of pain, distress, and discomfort in experimental animals described by Morton and Griffiths, except for temperature evaluation. Protocols were reviewed and approved by the Ethics Committee of the Universidad de Santiago de Chile.

## Author Contributions

JM, CC, CB, and DR: phagocytosis assay, antigen transference, and fusion experiments. SC, CR-P, XL, and ET: molecular biology. CB, CC, and CB-A: dendritic cell experiments. MI and MM: experimental molecular biology design. MC-SM, LR, and DS experimental immunological design. AE, RF, EL-S, DR, CR-P, and CA-C: experimental design and wrote the paper.

## Conflict of Interest Statement

The authors declare that the research was conducted in the absence of any commercial or financial relationships that could be construed as a potential conflict of interest.
